# (*S*)-(*Z*)-Methyl 2-[2,3-bis­(benzyl­oxy­carbon­yl)guanidino]-4-methyl­penta­no­ate

**DOI:** 10.1107/S1600536810050130

**Published:** 2010-12-04

**Authors:** Chris F. Fronczek, HyunJoo Kil, Mark L. McLaughlin, Frank R. Fronczek

**Affiliations:** a Department of Chemistry, Louisiana State University, Baton Rouge, LA 70803-1804, USA; b Department of Chemistry, University of South Florida, 4202 E. Fowler Avenue, CHE 205A, Tampa, FL 33620-5250, USA

## Abstract

The title mol­ecule, C_24_H_29_N_3_O_6_, has a nearly planar ten-atom C_3_N_3_O_4_ core, on account of both N—H groups forming six-membered-ring intra­molecular hydrogen bonds to carbamate carbonyl O atoms. The absolute configuration was determined from resonant scattering of light atoms in Mo *K*α radiation, agreeing with the configuration of starting materials.

## Related literature

For related structures, see: Travlos & White (1994 ▶); Feichtinger *et al.* (1998 ▶); Marsh (2002 ▶). For graph sets, see: Etter (1990 ▶). For absolute configuration based on resonant scattering from light atoms, see: Hooft *et al.* (2008 ▶); Fronczek (2010 ▶); Lutz & van Krieken (2010 ▶); Thompson *et al.* (2008 ▶).
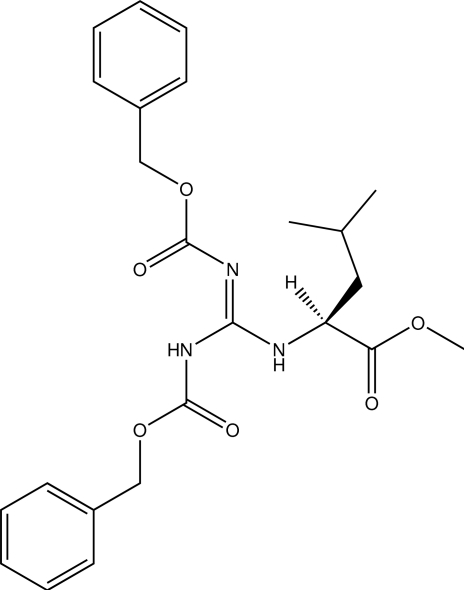


## Experimental

### 

#### Crystal data

C_24_H_29_N_3_O_6_
*M* *_r_* = 455.50
Orthorhombic, 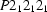

*a* = 7.7203 (5) Å
*b* = 14.2043 (10) Å
*c* = 21.280 (2) Å
*V* = 2333.6 (3) Å^3^
*Z* = 4
Mo *K*α radiation
μ = 0.09 mm^−1^
*T* = 90 K
0.30 × 0.28 × 0.15 mm

#### Data collection

Nonius KappaCCD diffractometer with an Oxford Cryosystems Cryostream cooler
43001 measured reflections
10411 independent reflections
9219 reflections with *I* > 2σ(*I*)
*R*_int_ = 0.056

#### Refinement

*R*[*F* ^2^ > 2σ(*F* ^2^)] = 0.043
*wR*(*F* ^2^) = 0.101
*S* = 1.02
10411 reflections
307 parameters
H atoms treated by a mixture of independent and constrained refinement
Δρ_max_ = 0.33 e Å^−3^
Δρ_min_ = −0.28 e Å^−3^
Absolute structure: Flack (1983 ▶), 4545 Friedel pairs
Flack parameter: 0.2 (5)

### 

Data collection: *COLLECT* (Nonius, 2000 ▶); cell refinement: *SCALEPACK* (Otwinowski & Minor, 1997 ▶); data reduction: *DENZO* (Otwinowski & Minor, 1997 ▶) and *SCALEPACK*; program(s) used to solve structure: *SIR97* (Altomare *et al.*, 1999 ▶); program(s) used to refine structure: *SHELXL97* (Sheldrick, 2008 ▶); molecular graphics: *ORTEP-3 for Windows* (Farrugia, 1997 ▶); software used to prepare material for publication: *SHELXL97*.

## Supplementary Material

Crystal structure: contains datablocks global, I. DOI: 10.1107/S1600536810050130/om2385sup1.cif

Structure factors: contains datablocks I. DOI: 10.1107/S1600536810050130/om2385Isup2.hkl

Additional supplementary materials:  crystallographic information; 3D view; checkCIF report

## Figures and Tables

**Table 1:** Hydrogen-bond geometry (Å, °)

*D*—H⋯*A*	*D*—H	H⋯*A*	*D*⋯*A*	*D*—H⋯*A*
N1—H1*N*⋯O5	0.849 (16)	2.051 (16)	2.7047 (11)	133.3 (14)
N3—H3*N*⋯O4	0.873 (16)	1.898 (16)	2.6306 (11)	140.4 (14)

**Fig. 1.**
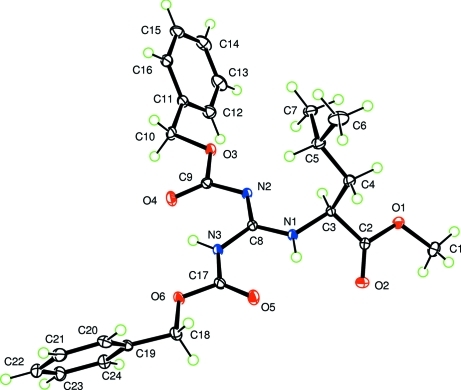
Ellipsoids at the 50% level, with H atoms having arbitrary radius.

## supplementary crystallographic information

### Comment

Molecules used as drugs frequently contain heterocyclic subunits, and substituted guanidine or amidine compounds are very important intermediates in the synthesis of many heterocyclic compounds. However, substituted guanidines and amidine compounds themselves can be difficult to synthesize. Thus, synthesis of substituted guanidines is important and interesting. One possible route to these compounds is to use 1,3-bis(benzyloxycarbonyl)-2-methyl-2-thiopseudourea, which has a good leaving group and *L*-leucine methyl ester hydrochloride, which is a good nucleophile. Reaction of these starting materials led to successful synthesis of the chiral title compound, which was confirmed by crystal structure determination.

The structure, shown in Figure 1, has a guanidine at its core. The three C—N distances of the guanidine vary from 1.3225 (12) to 1.3864 (12) Å, with the shortest being the formal double bond to the unprotonated N atom N2 and the longest being to the other carbamate N atom N3. These values are in good agreement with those seen in 1,2-bis(methoxycarbonyl)-3-phenylguanidine, (Travlos & White, 1994), in which the length pattern is the same and the range of lengths is 1.309 (3) to 1.388 (4) Å. In *N*,*N*',*N*''-tris(t-butoxycarbonyl)guanidine (Feichtinger *et al.*, 1998; space group corrected by Marsh, 2002), the C═N and C—NH groups are disordered, and the C—N distance is 1.343 Å. In the title compound, the two N—H groups form intramolecular hydrogen bonds with graph set (Etter, 1990) S(6). The hydrogen bonding leads to a fairly planar central C_3_N_3_O_4_ portion of the molecule, which has a mean deviation 0.019 Å from coplanarity and a maximum deviation 0.0533 (10) Å for N3.

The lone stereocenter is carbon C3, with (S) configuration, as known from starting material *L*-leucine. Absolute configuration determination based on resonant scattering of the light atoms in Mo *K*α radiation was possible for this structure, on account of the excellent quality of the crystal, the fact that it is relatively rich in O and N, the high resolution of the data, and the completeness of the set of 4545 Bijvoet pairs, which were kept separate in the refinement. While the Flack (1983) parameter is unconvincing, with a value of 0.2 (5), the Hooft *et al.* (2008) parameter *y* = 0.0 (2) has a much smaller uncertainty, and the Hooft P2(true) value is 1.000. A number of oxygen-rich compounds producing Mo data sets of similar high quality have been shown to yield similarly reliable absolute-structure results, agreeing with the known configurations (Fronczek, 2010; Lutz & van Krieken, 2010; Thompson *et al.*, 2008).

### Experimental

A mixture of 1,3-bis(benzyloxycarbonyl)-2-methyl-2-thiopseudourea (2.79 mmol, 1 g), *L*-leucine methyl ester hydrochloride (2.79 mmol, 0.51 g), and triethylamine (2.79 mmole, 0.4 ml) in THF (absolute, 10 mL) was stirred at 338 K. The mixture was brought to room temperature, and the precipitate was filtered by vacuum. After evaporation of all solvents from the filtrate, the product was purified by chromatography (EtOAc/hexane, 1:4). The product was isolated as colorless crystals in 46% yield. ^1^H NMR (Methanol, 400 MHz): δ 0.85–0.88 (dd, 6H), 1.58–1.60 (m, 2H), 1.67–1.74 (m, 1H), 3.63 (s, 3H), 4.54–4.59 (m, 1H), 7.28–7.43 (m, 10H), 8.53–8.55 (d, 1H), 11.47 (s, 1H). ^13^C NMR (Methanol, 400 MHz): δ 22.12, 24.95, 67.08, 68.47, 128.44, 128.54, 128.93, 129.01, 129.13, 155.36, 163.12, 172.39. MS m/*z* 456.21 [*M*+H]^+^, 478.19 [*M*+Na]^+^.

### Refinement

All H atoms were visible in difference maps, and those on C were placed in idealized positions with C—H distances 0.95 - 1.00 Å and thereafter treated as riding. Coordinates for the H atoms on N were refined. *U*_iso_ for H was assigned as 1.2 times *U*_eq_ of the attached atoms (1.5 for methyl). A torsional parameter was refined for each methyl group. Friedel pairs were kept separate in the refinement.

### Crystal data

**Table:**

C_24_H_29_N_3_O_6_	*F*(000) = 968
*M**_r_* = 455.50	*D*_x_ = 1.297 Mg m^−^^3^
Orthorhombic, *P*2_1_2_1_2_1_	Mo *K*α radiation, λ = 0.71073 Å
Hall symbol: P 2ac 2ab	Cell parameters from 5760 reflections
*a* = 7.7203 (5) Å	θ = 2.5–36.1°
*b* = 14.2043 (10) Å	µ = 0.09 mm^−^^1^
*c* = 21.280 (2) Å	*T* = 90 K
*V* = 2333.6 (3) Å^3^	Fragment, colourless
*Z* = 4	0.30 × 0.28 × 0.15 mm

### Data collection

**Table:**

Nonius KappaCCD (with an Oxford Cryosystems Cryostream cooler) diffractometer	9219 reflections with *I* > 2σ(*I*)
Radiation source: fine-focus sealed tube	*R*_int_ = 0.056
graphite	θ_max_ = 36.1°, θ_min_ = 2.8°
ω and φ scans	*h* = −12→12
43001 measured reflections	*k* = −22→22
10411 independent reflections	*l* = −34→34

### Refinement

**Table:**

Refinement on *F*^2^	Secondary atom site location: difference Fourier map
Least-squares matrix: full	Hydrogen site location: inferred from neighbouring sites
*R*[*F*^2^ > 2σ(*F*^2^)] = 0.043	H atoms treated by a mixture of independent and constrained refinement
*wR*(*F*^2^) = 0.101	*w* = 1/[σ^2^(*F*_o_^2^) + (0.0473*P*)^2^ + 0.6349*P*] where *P* = (*F*_o_^2^ + 2*F*_c_^2^)/3
*S* = 1.02	(Δ/σ)_max_ = 0.001
10411 reflections	Δρ_max_ = 0.33 e Å^−^^3^
307 parameters	Δρ_min_ = −0.28 e Å^−^^3^
0 restraints	Absolute structure: Flack (1983), 4545 Friedel pairs
Primary atom site location: structure-invariant direct methods	Flack parameter: 0.2 (5)

### Special details

**Table:**

Geometry. All e.s.d.'s (except the e.s.d. in the dihedral angle between two l.s. planes) are estimated using the full covariance matrix. The cell e.s.d.'s are taken into account individually in the estimation of e.s.d.'s in distances, angles and torsion angles; correlations between e.s.d.'s in cell parameters are only used when they are defined by crystal symmetry. An approximate (isotropic) treatment of cell e.s.d.'s is used for estimating e.s.d.'s involving l.s. planes.
Refinement. Refinement of *F*^2^ against ALL reflections. The weighted *R*-factor *wR* and goodness of fit *S* are based on *F*^2^, conventional *R*-factors *R* are based on *F*, with *F* set to zero for negative *F*^2^. The threshold expression of *F*^2^ > σ(*F*^2^) is used only for calculating *R*-factors(gt) *etc*. and is not relevant to the choice of reflections for refinement. *R*-factors based on *F*^2^ are statistically about twice as large as those based on *F*, and *R*- factors based on ALL data will be even larger.

### Fractional atomic coordinates and isotropic or equivalent isotropic displacement parameters (Å^2^)

**Table:**

	*x*	*y*	*z*	*U*_iso_*/*U*_eq_	
O1	0.58734 (12)	0.48105 (6)	0.79229 (4)	0.01853 (16)	
O2	0.69349 (11)	0.37173 (6)	0.72588 (4)	0.01797 (15)	
O3	0.66892 (11)	0.75163 (5)	0.55412 (3)	0.01520 (14)	
O4	0.70303 (12)	0.64048 (5)	0.47943 (4)	0.01833 (15)	
O5	0.57441 (13)	0.31412 (5)	0.54332 (4)	0.02052 (17)	
O6	0.65667 (11)	0.35908 (5)	0.44569 (3)	0.01435 (13)	
N1	0.55713 (12)	0.46022 (6)	0.62459 (4)	0.01239 (14)	
H1N	0.555 (2)	0.4008 (11)	0.6200 (7)	0.015*	
N2	0.62092 (12)	0.60509 (5)	0.58336 (4)	0.01166 (14)	
N3	0.63981 (13)	0.46856 (6)	0.51925 (4)	0.01316 (15)	
H3N	0.665 (2)	0.5094 (11)	0.4900 (7)	0.016*	
C1	0.66331 (18)	0.43149 (9)	0.84503 (5)	0.0225 (2)	
H1A	0.6032	0.3714	0.8511	0.034*	
H1B	0.6519	0.4699	0.8831	0.034*	
H1C	0.7862	0.4197	0.8366	0.034*	
C2	0.61124 (13)	0.44220 (7)	0.73573 (4)	0.01164 (15)	
C3	0.51657 (13)	0.49959 (7)	0.68598 (4)	0.01065 (15)	
H3	0.5592	0.5660	0.6876	0.013*	
C4	0.32033 (13)	0.49892 (7)	0.69888 (4)	0.01355 (16)	
H4A	0.2779	0.4334	0.6954	0.016*	
H4B	0.3005	0.5198	0.7427	0.016*	
C5	0.21252 (14)	0.56144 (7)	0.65481 (5)	0.01525 (17)	
H5	0.2404	0.5437	0.6105	0.018*	
C6	0.01921 (16)	0.54251 (9)	0.66614 (7)	0.0256 (2)	
H6A	−0.0103	0.5586	0.7096	0.038*	
H6B	−0.0055	0.4758	0.6587	0.038*	
H6C	−0.0499	0.5811	0.6374	0.038*	
C7	0.25316 (14)	0.66599 (7)	0.66332 (5)	0.01623 (18)	
H7A	0.1775	0.7033	0.6360	0.024*	
H7B	0.3744	0.6778	0.6522	0.024*	
H7C	0.2336	0.6839	0.7072	0.024*	
C8	0.60582 (13)	0.51301 (6)	0.57593 (4)	0.01072 (15)	
C9	0.66760 (13)	0.66107 (7)	0.53418 (4)	0.01203 (15)	
C10	0.70649 (16)	0.82125 (7)	0.50644 (5)	0.01594 (18)	
H10A	0.6129	0.8227	0.4747	0.019*	
H10B	0.8168	0.8061	0.4850	0.019*	
C11	0.71972 (14)	0.91503 (7)	0.53912 (5)	0.01436 (17)	
C12	0.83270 (16)	0.92721 (8)	0.58972 (5)	0.01901 (19)	
H12	0.9022	0.8761	0.6036	0.023*	
C13	0.84373 (17)	1.01415 (9)	0.61988 (6)	0.0235 (2)	
H13	0.9205	1.0221	0.6544	0.028*	
C14	0.74245 (17)	1.08929 (8)	0.59950 (6)	0.0243 (2)	
H14	0.7502	1.1485	0.6201	0.029*	
C15	0.63033 (17)	1.07768 (7)	0.54924 (6)	0.0220 (2)	
H15	0.5616	1.1290	0.5353	0.026*	
C16	0.61826 (16)	0.99065 (7)	0.51914 (5)	0.01785 (18)	
H16	0.5406	0.9828	0.4849	0.021*	
C17	0.61893 (14)	0.37440 (7)	0.50618 (5)	0.01343 (16)	
C18	0.63343 (15)	0.26163 (7)	0.42515 (5)	0.01528 (18)	
H18A	0.5126	0.2412	0.4326	0.018*	
H18B	0.7119	0.2194	0.4489	0.018*	
C19	0.67470 (14)	0.25779 (6)	0.35627 (4)	0.01216 (15)	
C20	0.55057 (14)	0.22770 (7)	0.31311 (5)	0.01544 (17)	
H20	0.4373	0.2120	0.3270	0.019*	
C21	0.59258 (16)	0.22057 (8)	0.24956 (5)	0.0188 (2)	
H21	0.5084	0.1988	0.2204	0.023*	
C22	0.75696 (16)	0.24510 (8)	0.22862 (5)	0.0190 (2)	
H22	0.7849	0.2410	0.1852	0.023*	
C23	0.88041 (15)	0.27570 (7)	0.27170 (5)	0.01767 (18)	
H23	0.9928	0.2928	0.2576	0.021*	
C24	0.84025 (14)	0.28140 (7)	0.33530 (5)	0.01503 (17)	
H24	0.9257	0.3014	0.3645	0.018*	

### Atomic displacement parameters (Å^2^)

**Table:**

	*U*^11^	*U*^22^	*U*^33^	*U*^12^	*U*^13^	*U*^23^
O1	0.0276 (4)	0.0184 (3)	0.0095 (3)	0.0087 (3)	−0.0024 (3)	−0.0005 (3)
O2	0.0190 (4)	0.0179 (3)	0.0170 (3)	0.0076 (3)	0.0007 (3)	0.0004 (3)
O3	0.0251 (4)	0.0083 (3)	0.0122 (3)	−0.0017 (3)	0.0044 (3)	0.0002 (2)
O4	0.0303 (4)	0.0128 (3)	0.0120 (3)	−0.0029 (3)	0.0067 (3)	−0.0006 (2)
O5	0.0370 (5)	0.0108 (3)	0.0137 (3)	−0.0018 (3)	0.0072 (3)	−0.0001 (3)
O6	0.0222 (4)	0.0101 (3)	0.0107 (3)	−0.0015 (3)	0.0039 (3)	−0.0022 (2)
N1	0.0194 (4)	0.0083 (3)	0.0095 (3)	−0.0002 (3)	0.0026 (3)	−0.0005 (2)
N2	0.0163 (4)	0.0084 (3)	0.0103 (3)	−0.0011 (3)	0.0016 (3)	0.0001 (2)
N3	0.0209 (4)	0.0087 (3)	0.0099 (3)	−0.0009 (3)	0.0035 (3)	−0.0003 (2)
C1	0.0282 (6)	0.0277 (5)	0.0115 (4)	0.0081 (5)	−0.0040 (4)	0.0028 (4)
C2	0.0121 (4)	0.0126 (4)	0.0103 (3)	−0.0008 (3)	0.0002 (3)	0.0011 (3)
C3	0.0141 (4)	0.0096 (3)	0.0083 (3)	0.0009 (3)	0.0012 (3)	0.0000 (3)
C4	0.0127 (4)	0.0144 (4)	0.0136 (4)	0.0011 (3)	0.0005 (3)	0.0016 (3)
C5	0.0156 (4)	0.0147 (4)	0.0154 (4)	0.0030 (3)	−0.0035 (3)	−0.0015 (3)
C6	0.0152 (5)	0.0239 (5)	0.0377 (7)	0.0013 (4)	−0.0054 (5)	−0.0014 (5)
C7	0.0177 (5)	0.0139 (4)	0.0171 (4)	0.0043 (3)	−0.0029 (3)	−0.0012 (3)
C8	0.0124 (4)	0.0105 (3)	0.0092 (3)	0.0006 (3)	0.0005 (3)	−0.0004 (3)
C9	0.0128 (4)	0.0100 (3)	0.0132 (4)	−0.0004 (3)	0.0006 (3)	0.0002 (3)
C10	0.0252 (5)	0.0099 (4)	0.0127 (4)	−0.0022 (3)	0.0047 (4)	0.0019 (3)
C11	0.0193 (4)	0.0094 (3)	0.0144 (4)	−0.0018 (3)	0.0053 (3)	0.0004 (3)
C12	0.0218 (5)	0.0158 (4)	0.0195 (4)	−0.0004 (4)	0.0029 (4)	−0.0013 (4)
C13	0.0238 (5)	0.0228 (5)	0.0239 (5)	−0.0051 (4)	0.0040 (4)	−0.0084 (4)
C14	0.0280 (6)	0.0147 (4)	0.0302 (6)	−0.0059 (4)	0.0126 (5)	−0.0070 (4)
C15	0.0289 (6)	0.0111 (4)	0.0259 (5)	0.0028 (4)	0.0118 (4)	0.0016 (4)
C16	0.0223 (5)	0.0137 (4)	0.0175 (4)	0.0019 (4)	0.0059 (4)	0.0024 (3)
C17	0.0180 (4)	0.0108 (4)	0.0115 (4)	0.0009 (3)	0.0024 (3)	−0.0022 (3)
C18	0.0231 (5)	0.0096 (4)	0.0132 (4)	−0.0026 (3)	0.0042 (3)	−0.0027 (3)
C19	0.0155 (4)	0.0097 (3)	0.0113 (3)	0.0005 (3)	0.0013 (3)	−0.0017 (3)
C20	0.0140 (4)	0.0146 (4)	0.0178 (4)	0.0018 (3)	−0.0006 (3)	−0.0039 (3)
C21	0.0219 (5)	0.0186 (4)	0.0158 (4)	0.0056 (4)	−0.0070 (4)	−0.0041 (4)
C22	0.0287 (6)	0.0165 (4)	0.0118 (4)	0.0045 (4)	0.0010 (4)	0.0008 (3)
C23	0.0199 (5)	0.0169 (4)	0.0163 (4)	−0.0009 (4)	0.0045 (4)	0.0015 (3)
C24	0.0163 (4)	0.0150 (4)	0.0138 (4)	−0.0025 (3)	0.0005 (3)	−0.0005 (3)

### Geometric parameters (Å, °)

**Table:**

O1—C2	1.3369 (12)	C7—H7A	0.9800
O1—C1	1.4488 (13)	C7—H7B	0.9800
O2—C2	1.2037 (12)	C7—H7C	0.9800
O3—C9	1.3546 (12)	C10—C11	1.5061 (14)
O3—C10	1.4462 (12)	C10—H10A	0.9900
O4—C9	1.2321 (12)	C10—H10B	0.9900
O5—C17	1.2147 (12)	C11—C16	1.3958 (15)
O6—C17	1.3377 (12)	C11—C12	1.3966 (16)
O6—C18	1.4626 (12)	C12—C13	1.3943 (16)
N1—C8	1.3326 (12)	C12—H12	0.9500
N1—C3	1.4550 (12)	C13—C14	1.3923 (19)
N1—H1N	0.849 (16)	C13—H13	0.9500
N2—C8	1.3225 (12)	C14—C15	1.386 (2)
N2—C9	1.3628 (12)	C14—H14	0.9500
N3—C17	1.3756 (12)	C15—C16	1.3954 (15)
N3—C8	1.3864 (12)	C15—H15	0.9500
N3—H3N	0.873 (16)	C16—H16	0.9500
C1—H1A	0.9800	C18—C19	1.5011 (14)
C1—H1B	0.9800	C18—H18A	0.9900
C1—H1C	0.9800	C18—H18B	0.9900
C2—C3	1.5230 (13)	C19—C20	1.3944 (14)
C3—C4	1.5397 (14)	C19—C24	1.3947 (15)
C3—H3	1.0000	C20—C21	1.3944 (16)
C4—C5	1.5366 (14)	C20—H20	0.9500
C4—H4A	0.9900	C21—C22	1.3895 (18)
C4—H4B	0.9900	C21—H21	0.9500
C5—C7	1.5286 (15)	C22—C23	1.3920 (17)
C5—C6	1.5355 (17)	C22—H22	0.9500
C5—H5	1.0000	C23—C24	1.3910 (15)
C6—H6A	0.9800	C23—H23	0.9500
C6—H6B	0.9800	C24—H24	0.9500
C6—H6C	0.9800		
			
C2—O1—C1	116.17 (8)	O4—C9—N2	130.27 (9)
C9—O3—C10	115.54 (8)	O3—C9—N2	108.40 (8)
C17—O6—C18	114.50 (8)	O3—C10—C11	107.12 (8)
C8—N1—C3	122.83 (8)	O3—C10—H10A	110.3
C8—N1—H1N	118.5 (11)	C11—C10—H10A	110.3
C3—N1—H1N	118.6 (11)	O3—C10—H10B	110.3
C8—N2—C9	120.56 (8)	C11—C10—H10B	110.3
C17—N3—C8	126.62 (8)	H10A—C10—H10B	108.5
C17—N3—H3N	121.8 (10)	C16—C11—C12	119.34 (10)
C8—N3—H3N	111.1 (10)	C16—C11—C10	120.13 (10)
O1—C1—H1A	109.5	C12—C11—C10	120.53 (10)
O1—C1—H1B	109.5	C13—C12—C11	120.18 (11)
H1A—C1—H1B	109.5	C13—C12—H12	119.9
O1—C1—H1C	109.5	C11—C12—H12	119.9
H1A—C1—H1C	109.5	C14—C13—C12	120.09 (12)
H1B—C1—H1C	109.5	C14—C13—H13	120.0
O2—C2—O1	124.94 (9)	C12—C13—H13	120.0
O2—C2—C3	125.25 (9)	C15—C14—C13	119.99 (11)
O1—C2—C3	109.80 (8)	C15—C14—H14	120.0
N1—C3—C2	108.37 (8)	C13—C14—H14	120.0
N1—C3—C4	111.68 (8)	C14—C15—C16	120.10 (11)
C2—C3—C4	110.18 (8)	C14—C15—H15	120.0
N1—C3—H3	108.9	C16—C15—H15	120.0
C2—C3—H3	108.9	C15—C16—C11	120.30 (11)
C4—C3—H3	108.9	C15—C16—H16	119.9
C5—C4—C3	114.87 (8)	C11—C16—H16	119.9
C5—C4—H4A	108.5	O5—C17—O6	124.96 (9)
C3—C4—H4A	108.5	O5—C17—N3	125.94 (9)
C5—C4—H4B	108.5	O6—C17—N3	109.10 (8)
C3—C4—H4B	108.5	O6—C18—C19	107.47 (8)
H4A—C4—H4B	107.5	O6—C18—H18A	110.2
C7—C5—C6	110.55 (9)	C19—C18—H18A	110.2
C7—C5—C4	112.21 (8)	O6—C18—H18B	110.2
C6—C5—C4	109.24 (9)	C19—C18—H18B	110.2
C7—C5—H5	108.2	H18A—C18—H18B	108.5
C6—C5—H5	108.2	C20—C19—C24	119.53 (9)
C4—C5—H5	108.2	C20—C19—C18	120.56 (9)
C5—C6—H6A	109.5	C24—C19—C18	119.88 (9)
C5—C6—H6B	109.5	C19—C20—C21	120.08 (10)
H6A—C6—H6B	109.5	C19—C20—H20	120.0
C5—C6—H6C	109.5	C21—C20—H20	120.0
H6A—C6—H6C	109.5	C22—C21—C20	120.35 (10)
H6B—C6—H6C	109.5	C22—C21—H21	119.8
C5—C7—H7A	109.5	C20—C21—H21	119.8
C5—C7—H7B	109.5	C21—C22—C23	119.50 (10)
H7A—C7—H7B	109.5	C21—C22—H22	120.2
C5—C7—H7C	109.5	C23—C22—H22	120.2
H7A—C7—H7C	109.5	C24—C23—C22	120.42 (11)
H7B—C7—H7C	109.5	C24—C23—H23	119.8
N2—C8—N1	119.24 (8)	C22—C23—H23	119.8
N2—C8—N3	122.53 (8)	C23—C24—C19	120.10 (10)
N1—C8—N3	118.24 (8)	C23—C24—H24	119.9
O4—C9—O3	121.32 (9)	C19—C24—H24	119.9
			
C1—O1—C2—O2	−1.94 (16)	O3—C10—C11—C12	54.29 (13)
C1—O1—C2—C3	177.18 (9)	C16—C11—C12—C13	0.00 (16)
C8—N1—C3—C2	−132.15 (10)	C10—C11—C12—C13	−179.77 (10)
C8—N1—C3—C4	106.31 (11)	C11—C12—C13—C14	−0.20 (18)
O2—C2—C3—N1	−5.64 (14)	C12—C13—C14—C15	0.08 (18)
O1—C2—C3—N1	175.24 (8)	C13—C14—C15—C16	0.24 (18)
O2—C2—C3—C4	116.82 (11)	C14—C15—C16—C11	−0.45 (17)
O1—C2—C3—C4	−62.30 (11)	C12—C11—C16—C15	0.33 (16)
N1—C3—C4—C5	−64.38 (11)	C10—C11—C16—C15	−179.91 (10)
C2—C3—C4—C5	175.13 (8)	C18—O6—C17—O5	2.47 (16)
C3—C4—C5—C7	−65.68 (11)	C18—O6—C17—N3	−177.91 (9)
C3—C4—C5—C6	171.35 (9)	C8—N3—C17—O5	−3.52 (19)
C9—N2—C8—N1	−178.87 (9)	C8—N3—C17—O6	176.87 (10)
C9—N2—C8—N3	1.53 (15)	C17—O6—C18—C19	177.95 (9)
C3—N1—C8—N2	1.03 (15)	O6—C18—C19—C20	−120.63 (10)
C3—N1—C8—N3	−179.35 (9)	O6—C18—C19—C24	61.55 (12)
C17—N3—C8—N2	−176.29 (10)	C24—C19—C20—C21	0.56 (15)
C17—N3—C8—N1	4.11 (16)	C18—C19—C20—C21	−177.26 (9)
C10—O3—C9—O4	2.71 (15)	C19—C20—C21—C22	−1.28 (16)
C10—O3—C9—N2	−176.60 (9)	C20—C21—C22—C23	0.85 (16)
C8—N2—C9—O4	−0.78 (18)	C21—C22—C23—C24	0.28 (16)
C8—N2—C9—O3	178.44 (9)	C22—C23—C24—C19	−0.99 (16)
C9—O3—C10—C11	−174.34 (9)	C20—C19—C24—C23	0.56 (15)
O3—C10—C11—C16	−125.47 (10)	C18—C19—C24—C23	178.40 (9)

### Hydrogen-bond geometry (Å, °)

**Table:**

*D*—H···*A*	*D*—H	H···*A*	*D*···*A*	*D*—H···*A*
N1—H1N···O5	0.849 (16)	2.051 (16)	2.7047 (11)	133.3 (14)
N3—H3N···O4	0.873 (16)	1.898 (16)	2.6306 (11)	140.4 (14)
